# Geriatric Depression and Inappropriate Medication: Benefits of Interprofessional Team Cooperation in Nursing Homes

**DOI:** 10.3390/ijerph182312438

**Published:** 2021-11-26

**Authors:** Hana Vankova, Iva Holmerova, Ladislav Volicer

**Affiliations:** 1 Third Faculty of Medicine, Charles University, 10000 Praha, Czech Republic; lvolicer@usf.edu; 2 Faculty of Humanities, CELLO-ILC-CZ, Charles University, 18200 Praha, Czech Republic; iva.holmerova@gerontocentrum.cz; 3 School of Aging Studies, University of South Florida, Tampa, FL 34639, USA

**Keywords:** depression, benzodiazepines, interprofessional team, nurse, older adults

## Abstract

An investigation of inappropriate medication use in treatment of depressivity in institutionalized older adults, based on a nurse-led evaluation of functional status and depressive symptoms in nursing home residents. **Methods:** A cross-sectional multicenter study was performed using records from 1087 residents cared for in fifteen nursing homes (NHs) in the Czech Republic. Inclusion criteria were being a permanent resident of one of the facilities, being 60 years of age or older, having a Geriatric Depression Scale score of 6 or more, and having a Mini Mental State examination score 10 or more. The final sample for analysis included 317 depressed NH residents. **Results:** 52 percent of NH residents with depressivity had no antidepressant treatment. Benzodiazepines were the only medication in 16 percent of depressed residents, and were added to antidepressant treatment in 18 percent of residents. Benzodiazepine users had significantly higher GDS scores compared to non-users (*p* = 0.007). **Conclusion:** More than half of depressed NH residents remained without antidepressant treatment. Residents inappropriately treated with benzodiazepines were more depressed than residents treated with antidepressants only, or even not treated at all. Cooperation of the interprofessional team in the screening of depressive symptoms has the potential to improve the quality of care.

## 1. Introduction

Depression is a mood disorder that causes persistent feelings of sadness, loss of interest, hopelessness, helplessness, and irritability [1]. Depression is common in nursing home residents, with prevalence ranging from 15 percent to up to 65 percent [1,2,3,4,5,6]. An important contributing factor is the high prevalence of depression in Alzheimer’s disease, caused by a serotoninergic deficit [7,8] which is more severe in patients who are more depressed [9].

Although depression is most commonly treated with selective serotonin reuptake inhibitors (SSRI), some persons with depression are treated with benzodiazepines [10]. More recently, Assem-Hilger found that the frequency of SSRI use for treatment of depression was similar to the frequency of the benzodiazepine use in a population of depressed community residents 75 years old and older [11]. Since then, several clinical practice guidelines stated that treatment of depression with benzodiazepines is not indicated [12,13,14]. In addition, use of benzodiazepines in older adults is not considered appropriate because of the increased risk of side effects [15] including falls, hip fracture, dementia, and mortality [16,17,18,19].

The question remains whether these guidelines have been followed by changes in real practice. The results of a ten-year follow-up in 12,556 nursing homes (NHs) in the United States observed increased anti-anxiety medication use increased from 15.5 percent to 18.5 percent [20]. There is a need for continual monitoring of benzodiazepine use in different countries [21], and the goal of our study responds to this need. The involvement of an interprofessional team in the detection of inappropriately treated geriatric depression is crucial in order to inspire daily practice in nursing homes. Our study brings to light new evidence regarding the population health aspects of ageing, with a focus on inappropriate medication use in geriatric depression.

## 2. Materials and Methods

A cross-sectional study was performed using records collected from 1087 nursing home residents cared for in fifteen nursing homes in the Czech Republic. All sixteen public nursing homes from one selected region of the Czech Republic were asked to participate; all of them agreed, however one of them was not able to cooperate due to reorganization in the nursing home, resulting in fifteen participating facilities. Inclusion criteria were (i) being a permanent resident of one of the facilities and (ii) being 60 years of age or older. In this study, the analysis focused on depressive NH residents as defined by the additional inclusion criteria (iii) a Geriatric Depression Scale (GDS) score of 6 or more, indicating presence of depression and (iv) a Mini Mental State examination (MMSE) score of 10 or more, to avoid the lower specificity of GDS results in people with more profound cognitive impairment [22,23]. Accordingly, 161 participants were not included in the analysis because of low or unknown MMSE scores, and 608 because of low GDS scores. Therefore, the final sample for the statistical analyses in the present study included 317 NHs residents. Data provided by the nursing homes were anonymous. The study was approved by the Ethical Committee and Internal Review Board at the Internal Grant Agency of the Ministry of Health of the Czech Republic.

The medication cards of nursing home residents were used for data collection. Medications were classified according to the Anatomical Therapeutic Chemical (ATC) classification system (WHO Collaborating Centre for Drug Statistics Methodology, 2016) into antidepressants (N06A) and benzodiazepines (N05BA, N05CD). According to the guidelines, every long-term use of benzodiazepines in geriatric patients is inappropriate [12,13,14]. An exception could be considered very short-term use for relief in people with panic disorder until the main therapy achieved full effect [24]. However, among our participants there were none diagnosed with panic disorder. Therefore, in this study every use of benzodiazepines in participants was considered inappropriate. Medical diagnoses of depression or anxiety disorder were made by a physician, mainly a psychiatrist or general practitioner. General practitioners were expected to visit these NHs at least weekly. Psychiatrists were visiting regularly, usually monthly. However, only some residents are seen by these physicians during their visits, mainly those presented by the nurses as those who needed an examination. Registered nurses administered the following tests:

- The Barthel Index [25] was used to measure functional ability to perform basic activities of daily living (ADLs; range 0–100, higher scores = better ability).
- Cognitive function was evaluated using the Mini-Mental State Examination scores, which were recorded in respondents’ clinical documentation [26], (range 0–30, higher scores = better cognition).
- Depressive symptoms were measured by the 15-item Geriatric Depression Scale [27], a screening scale for depression in older adults. The GDS scale consists of fifteen questions (e.g., ‘Do you feel that your life is empty?’) and has good reliability (Cronbach’s alpha 0.80; [28] and sufficient sensitivity (93 percent; [29]), even for those with cognitive deficits [30]. The GDS scale has been frequently used for NH residents [31,32]. The Czech language version, standardized for the use in the Czech Republic, was used [33].

The nurses also participated in a focus group where they could provide their reflection on the process of using above mentioned tests as well as on the role of a nurse in NHs. Field notes were taken during the meeting; recording was not taken. The authors of all quotes reproduced in this manuscript agreed with reproducing of their quotes anonymously, only with mentioning their job position (i.e., nurse in NH) [34].

### Statistical Analyses

Statistical Package for Social Sciences (IBM SPSS Statistics, Version 24.0; IBM Corporation, New York, NY, USA) was used to analyze the data. The normality of distribution was checked by Kolmogorov–Smirnov test where appropriate, in order to decide between use of t-tests or nonparametric tests in further analyses. Independent sample Mann–Whitney tests or chi-square tests were used to test differences between benzodiazepine users and non-users. In addition, general linear model univariate analysis (GLM univariate) was used to further analyze the results provided by independent sample Mann–Whitney tests. This procedure allows for comparison of four subgroups according to benzodiazepine and antidepressant use while testing for interaction and controlling for covariates. The two fixed factors design (benzodiazepine use; antidepressant use) was applied while controlling for NH location. Statistical significance for all statistical tests used was set at a two-tailed 0.05 level.

## 3. Results

### 3.1. Characteristics of the Sample

Characteristics of the sample and its subgroups according to their treatments are presented in Table 1. The mean age of the whole sample was 83 years. Most participants were women (73 percent), lived on an average 5.4 years in a nursing home and had 5.25 chronic diagnoses. They were dependent in ADLs and experienced some levels of cognitive impairment.

More than half (52 percent) of depressed NH residents remained without appropriate treatment. Benzodiazepines were the only medications in 16 percent of depressed residents. Almost half (48 percent) of the participants used antidepressants (AD) but still had depressive symptoms. Benzodiazepines (BZDs) were added to antidepressant treatment in 18 percent of residents (Table 1).

While the whole sample was defined by positive depressive symptoms (GDS ≥ 6), only 53 percent of residents had a documented diagnosis of depression and/or antidepressant treatment (medical diagnosis of depressive disorder was also explicitly recorded in 16 residents without antidepressant treatment). Only eight residents had a documented diagnosis of an anxiety disorder, and six of them were treated with BZDs.

### 3.2. Comparison of Participants Divided by Type of Treatment

Comparison of the characteristics of depressive NH residents using independent Mann–Whitney tests revealed that those treated with BZD had significantly higher depressive symptom scores than those without BZD treatment (Table 2). The distribution of the variables of age, ADL, GDS, and MMSE were checked for normality by Kolmogorov–Smirnov test, which resulted in the use of nonparametric Mann–Whitney tests for these variables. Residents with a documented diagnosis of anxiety disorder or depressive disorder were more likely to receive benzodiazepines. BZD users had a significantly higher load of depressive symptoms, and were predominantly female; there were no other significant differences between BZD users and non-users (Table 2). Comparing residents with and without antidepressant treatment, we found that participants treated with antidepressants had significantly lower ADL scores (AD group ADL 51.40 ± 30.48 vs. No AD group ADL 60.59 ± 30.29, *p* = 0.008). The participants with benzodiazepines added to antidepressants had a significantly higher load of depressive symptoms than those with antidepressants only (‘BZD added to AD group’ GDS = 9.55 ± 2.74 vs. ‘AD only group’ GDS = 8.46 ± 2.38; *p* = 0.013).

The results of GLM univariate analysis are presented in Table 3. In agreement with the results of the Mann–Whitney tests, benzodiazepine users had significantly higher GDS scores and were predominantly female, while other characteristics (age, cognitive status or dependency) were not related to BZD use. Antidepressant use was correlated with dependency and cognitive function, and was more common in highly dependent NH residents and in residents with more severe cognitive impairment. There was no significant interaction between BZD and antidepressants for any of the variables. GLM controlled for NH sites revealed that the results were not associated with specific NHs and the associations of gender and GDS scores with BZD use remained highly significant (gender *p* = 0.032; GDS score *p* = 0.006).

## 4. Discussion

This study focuses on important aspects of pharmacoepidemiology in nursing home residents based on 1087 residents, with 317 of them depressed. Our study confirms that benzodiazepine use may serve as an indicator of inappropriately treated geriatric depression in nursing home residents, which is consistent with a previous community-based study [11]. Residents treated with benzodiazepines deserve attention, as they had the highest load of depressive symptoms (displayed in Table 2 and Table 3).

The subgroup of residents who had symptoms of depression but were not diagnosed and had no pharmacotherapy and no psychotherapy could be characterized as those who were less disabled (Table 2 and Table 3). The reflection of the interprofessional team on the characteristics of residents with inappropriately treated depression is of high importance and may contribute to improvement in future.

During a focus group with nurses from these nursing homes about the outcomes, the nurses commented that “those who are bedbound we expect to be depressive and it is easier for us to understand their feelings”; “Those who can move better and are less dependent than the others here, we just did not expect to be depressive”; and “We refer those more disabled to the physician more often”. Another quotation described the process: “Physicians visit only those residents who ask to be seen or who are recommended for examination by the nurses” [34]. According to our previous study in NHs in the same region, there exist residents who were not seen by any physician for a year or even longer [35]. The role of a nurse in active detection of residents with inappropriately treated depression who need a physician’s intervention is essential. Therefore, the screening of depressive symptoms by nurses using standardized scales seems to be of high importance.

Our results also suggest that despite the published guidelines and the list of inappropriate medications including benzodiazepines [36], benzodiazepine misuse remains a problem in nursing homes. A focused study reported that in residents with at least one behavioral and psychological symptom of dementia, benzodiazepines and related drugs were used four times more frequently than antidepressants (AD 7.5 percent vs. BZD 28.5 percent; Ozaki et al., 2016).

As for the longitudinal trends, increasing use of benzodiazepines was reported in several countries. In the nursing home population, antianxiety medication use increased from 15.5 percent in 1996 to 18.5 percent in 2006, and a dramatic increase of antidepressant use (from 21.9 to 47.5 percent!) in this population in the same period did not prevent this [20]. Another study described trends of improvement in Ontario, Australia and the US Veteran population, while the overall prevalence of benzodiazepines still remained high, especially in the oldest age groups [37].

The trends in benzodiazepine use described above call into question the effectiveness of published articles and guidelines regarding prevention of the use of benzodiazepines in older adults with depression. The British review concludes that benzodiazepine prescription for unlicensed or unspecified indications (‘off-label’) is frequent, suggesting that “official recommendations concerning the use of these medications are widely ignored” [38]. According to the American Psychiatric Association guidelines, “benzodiazepines should not be the primary pharmacological agents for patients with major depressive disorder who have cooccurring anxiety symptoms” [14].

The comorbidity of depression and anxiety is found to be quite high [39], as risk factors for both may partially overlap [40,41]. However, even for pure generalized anxiety disorder (GAD), the first-choice treatment should be antidepressants and not benzodiazepines [42]. Moreover, benzodiazepines are considered an inappropriate medication in geriatric patients (Beers criteria [43]; STOPP criteria [44]; Choosing Wisely International campaign [45]). These principles are also reflected in the Czech guidelines for General Practitioners regarding depression treatment and inappropriate medication in geriatric patients [46,47]. Benzodiazepine use in our sample cannot be explained by the presence of anxiety disorders, as these disorders were diagnosed only in a very small number of residents. We could hypothesis that the intention was to enhance depression treatment by adding benzodiazepines to antidepressants in depressed residents. However, as described above, neither GAD nor depression with co-occurring anxiety symptoms are considered to be indications for benzodiazepines treatment, especially not in geriatric patients.

Reports on the characteristics of benzodiazepine users in NHs are quite sparse. A French study focused on NH structure- and organization-related indicators in relation to benzodiazepine prescriptions [48], and will be discussed further below. Brief information about pooled benzodiazepine prevalence in eight European countries is mentioned in the outcomes of the SHELTER project; benzodiazepines were used in 36 percent of 4023 European NH residents [5]. In a subgroup of residents with advanced cognitive impairment, benzodiazepines were used in 35.3 percent of residents, while antidepressants were used in 31.8 percent of a total sample of 1449 residents [49]. Both articles focus mostly on polypharmacy, and work with pooled data from all eight European countries.

Benzodiazepine users in our study did not differ from nonusers in age, cognition or dependency. Women used benzodiazepines significantly more frequently than men, which is consistent with other studies in Europe [11,50] and in the United States [6]. In those residents who had a documented diagnosis of depressive disorder, BZD treatment was even more frequent than in those who did not have documented diagnosis of depression. Thus, an appropriate diagnosis of depression unfortunately did not prevent BZD misuse in our sample. The results of our study correspond with the conclusions of Lai et al., who concluded that formulating guidelines for benzodiazepine use was not effective enough and recommended more complex interventions. [21]. Complex interventions are also recommended by a recent analysis of mental health care in older adults [51].

Reduction of inappropriate benzodiazepine prescription is a difficult process, even though several authors report partial successes using tapering protocols in benzodiazepine users supported by education of benzodiazepine users and/or physicians [52,53,54,55,56,57,58]. A French study concluded that a non-specific intervention designed to improve overall NH quality indicators through education and support of NH staff did not decrease benzodiazepine use [59]. This study also reported that none of the structural and organizational NH-related variables predicted either discontinuation or new use of benzodiazepines, contrary to the previous hypothesis [48,59].

Our study has several limitations. First, it used the GDS for people with cognitive impairment. However, this scale has been used in other studies [32], and in our previous study we found that GDS effectively described the affective status of NH residents with MMSE scores between 10 and 15 [23]. Second, we did not administer an anxiety scale to the participants; however, we had documented clinical diagnoses of anxiety disorder, which were quite rare. Finally, our study was cross-sectional, and therefore cannot assess causality. Interestingly, our results are in agreement with the results of a longitudinal study which found that benzodiazepine use concurrent with AD treatment does not significantly improve depressive outcomes in older veterans [60].

In this study, we observed a subgroup of residents who had depressive symptoms despite antidepressant treatment. Future research should focus on the question of why antidepressant treatment was not more effective, including the question of holistic mental care models [51]. While prevalence of benzodiazepine users among NH residents remains high, and discontinuation of benzodiazepines in chronic users is very difficult [59], preventive strategies focused on the motivation of residents, physicians and nursing home interprofessional team members to avoid onset of benzodiazepine treatment should deserve more attention.

Nursing homes are considered a special environment, where multiple actors are responsible for medication use. Inappropriate drug use is one of key factors worsening outcomes in long-term care [61,62]. For preventive strategies, it is essential to include all relevant members of the NH team (nurses, general practitioners, physicians, pharmacists and managers), together with residents and their families, to support motivation, education and willingness to change the practice of benzodiazepine misuse [57].

## 5. Conclusions

More than half (52 percent) of depressed NH residents remained without appropriate antidepressant treatment. Benzodiazepines are inappropriately used in NH residents with depressive symptoms, either as the only medication or added to antidepressant treatments. Residents treated with benzodiazepines were more depressed than residents treated only with antidepressants, and even those not treated at all. Nurse-led screening of depressive symptoms has good potential to improve the quality of care.

## Figures and Tables

**Table 1:** Characteristics of the sample (means ± SD, or %(*n*)).

	Sample	GDS	MMSE	ADL	Age	Gender (Female)	Clinical dg Depression	Clinical dg Anxiety
Total	100% (317)	8.77 ± 2.36	19.5 ± 5.87	56.7 ± 30.68	83.45 ± 7.71	73% (231)	53% (168)	3% (8)
Untreated depression	36% (114)	8.58 ± 2.05	19.18 ± 5.91	61.05 ± 29.36	83.3 ± 7.62	65% (74)	3% (9)	0% (0)
BZD only	16% (51)	8.98 ± 2.38	18.38 ± 5.80	59.48 ± 32.70	82.56 ± 8.45	81% (41)	2% (7)	0.5% (2)
BZD + AD	18% (57)	9.55 ± 2.74	20.54 ± 6.02	58.36 ± 33.03	83.18 ± 7.23	80% (46)	18% (57)	1% (4)
AD only	30% (95)	8.46 ± 2.38	19.88 ± 5.73	47.37 ± 28.31	84.26 ± 7.73	74% (70)	30% (95)	0.5% (2)

BZD = benzodiazepines. AD = antidepressants, ADL = activities of daily living, GDS = Geriatric Depression Scale, dg = diagnosis.

**Table 2:** Comparison of characteristics of depressive NH residents with and without benzodiazepine (BZD) treatment (*n* = 317, means ± SD; or %).

	Without BZD	With BZD	*p*
Age	83.74 ± 7.67	82.89 ± 7.79	0.271
Gender (female %)	68.7%	80.8%	0.028
Number of chronic diagnoses	5.16 ± 2.12	5.39 ± 2.40	0.666
MMSE	19.50 ± 5.83	19.52 ± 5.99	0.999
ADL	54.83 ± 29.6	58.88 ± 32.70	0.194
GDS	8.52 ± 2.20	9.28 ± 2.58	0.015
Diagnosis D (yes%)	61.6%	73.6%	0.022
Diagnosis A (yes%)	0.5%	1.9%	0.019

Independent samples Mann Whitney test or chi-square test used to compare both groups. Diagnosis D: diagnosis of depressive disorder and/or antidepressant prescription in medical documentation. Diagnosis A: diagnosis of anxiety disorder in medical documentation. ADL = activities of daily living. GDS = Geriatric Depression Scale.

**Table 3:** GLM univariate analysis: Benzodiazepine users compared with antidepressant users.

	BZD Users		AD Users		BZD x AD	
	F	*p*	F	*p*	F	*p*
Age	0.981	0.323	0.730	0.394	0.034	0.855
Gender	4.211	0.041	0.566	0.453	0.827	0.364
ADL	1.663	0.198	4.104	0.044	2.960	0.086
MMSE	0.010	0.919	4.144	0.043	1.094	0.296
GDS	7.348	0.007	0.702	0.403	1.492	0.223

BZD = benzodiazepines, AD = antidepressants, ADL = activities of daily living, GDS = Geriatric Depression Scale.

## Data Availability

The data that support the findings of this study are available from the project investigators but restrictions apply to the availability of these data, which were used under license for the current study. Data are however available from the authors upon reasonable request and with permission of all participating institutions.

## References

[1] Thakur M., Blazer D.G. (2008). Depression in Long-Term Care. J. Am. Med. Dir. Assoc..

[2] Tsai Y.-F., Chung J., Wong T.K.S., Huang C.-M. (2005). Comparison of the prevalence and risk factors for depressive symptoms among elderly nursing home residents in Taiwan and Hong Kong. Int. J. Geriatr. Psychiatry.

[3] Olin J.T., Schneider L.S., Katz I.R., Meyers B.S., Alexopoulos G.S., Breitner J.C., Bruce M.L., Caine E.D., Cummings J.L., Devanand D.P. (2002). Provisional Diagnostic Criteria for Depression of Alzheimer Disease. Am. J. Geriatr. Psychiatry.

[4] Volicer L., Frijters D., van der Steen J. (2011). Underdiagnosis and undertreatment of depression in nursing home residents. Eur. Geriatr. Med..

[5] Onder G., Liperoti R., Bernabei R., Landi F., Project F.T.S., Fialova D., Topinkova E., Tosato M., Danese P., Gallo P.F. (2012). Polypharmacy in Nursing Home in Europe: Results from the SHELTER Study. J. Gerontol. Ser. A Boil. Sci. Med. Sci..

[6] Giovannini S., Onder G., van der Roest H.G., Topinkova E., Gindin J., Cipriani M.C., Denkinger M.D., Bernabei R., Liperoti R., on behalf of the SHELTER Study Investigators (2020). Use of antidepressant medications among older adults in European long-term care facilities: A cross-sectional analysis from the SHELTER study. BMC Geriatr..

[7] Yamamoto T., Hirano A. (1985). Nucleus raphe dorsalis in Alzheimer’s disease: Neurofibrillary tangles and loss of large neurons. Ann. Neurol..

[8] Chen K.H., Reese E.A., Kim H.-W., Rapoport S.I., Rao J.S. (2011). Disturbed Neurotransmitter Transporter Expression in Alzheimer’s Disease Brain. J. Alzheimer’s Dis..

[9] Zweig R.M., Ross C.A., Hedreen J.C., Steele C., Cardillo J.E., Whitehouse P.J., Folstein M.F., Price D.L. (1988). The neuropathology of aminergic nuclei in Alzheimer’s disease. Ann. Neurol..

[10] Steffens D.C., Skoog I., Norton M.C., Hart A.D., Tschanz J.T., Plassman B.L., Wyse B.W., Welsh-Bohmer K.A., Breitner J.C. (2000). Prevalence of depression and its treatment in an elderly population: The Cache County study. Arch. Gen. Psychiatry.

[11] Assem-Hilger E., Jungwirth S., Weissgram S., Kirchmeyr W., Fischer P., Barnas C. (2009). Benzodiazepine use in the elderly: An indicator for inappropriately treated geriatric depression?. Int. J. Geriatr. Psychiatry.

[12] Alexopoulos G.S. (2011). Pharmacotherapy for Late-Life Depression. J. Clin. Psychiatry.

[13] O’Mahony D., O’Sullivan D., Byrne S., O’Connor M.N., Ryan C., Gallagher P. (2014). STOPP/START criteria for potentially inappropriate prescribing in older people: Version 2. Age Ageing.

[14] Gelenberg A.J., Freeman M.P., Markowitz J.C., Rosenbaum J.F., Thase M.E., Trivedi M.H., Silbersweig D.A. (2010). American Psychiatric Association. Practice Guideline for the Treatment of Patients with Major Depressive Disorder: Third Edition. Am. J. Psychiatry.

[15] Dunn R.L., Harrison N., Ripley T.L. (2011). The Beers Criteria as an Outpatient Screening Tool for Potentially Inappropriate Medications. Consult. Pharm..

[16] Cumming R.G., Le Couteur D.G., Le Conteur D.G. (2003). Benzodiazepines and Risk of Hip Fractures in Older People. CNS Drugs.

[17] De Gage S.B., Bégaud B., Bazin F., Verdoux H., Dartigues J.-F., Peres K., Kurth T., Pariente A. (2012). Benzodiazepine use and risk of dementia: Prospective population based study. BMJ.

[18] Weich S., Pearce H.L., Croft P., Singh S., Crome I., Bashford J., Frisher M. (2014). Effect of anxiolytic and hypnotic drug prescriptions on mortality hazards: Retrospective cohort study. BMJ.

[19] Olazarán J., Valle D., Serra J.A., Cano P., Muñiz R. (2013). Psychotropic Medications and Falls in Nursing Homes: A Cross-Sectional Study. J. Am. Med. Dir. Assoc..

[20] Hanlon J.T., Handler S.M., Castle N.G. (2010). Antidepressant Prescribing in US Nursing Homes between 1996 and 2006 and Its Relationship to Staffing Patterns and Use of Other Psychotropic Medications. J. Am. Med. Dir. Assoc..

[21] Lai I.-C., Wang M.-T., Wu B.-J., Wu H.-H., Lian P.-W. (2011). The use of benzodiazepine monotherapy for major depression before and after implementation of guidelines for benzodiazepine use. J. Clin. Pharm. Ther..

[22] McGivney S.A., Mulvihill M., Taylor B. (1994). Validating the GDS Depression Screen in the Nursing Home. J. Am. Geriatr. Soc..

[23] Vankova H., Holmerova I., Machacova K., Volicer L., Veleta P., Celko A.M. (2014). The Effect of Dance on Depressive Symptoms in Nursing Home Residents. J. Am. Med. Dir. Assoc..

[24] Susman J., Klee B. (2005). The Role of High-Potency Benzodiazepines in the Treatment of Panic Disorder. Prim. Care Companion J. Clin. Psychiatry.

[25] Mahoney F.I. (1965). Functional evaluation: The Barthel Index. Md. State Med. J..

[26] Folstein M.F., Folstein S.E., McHugh P.R. (1975). “Mini-mental state”: A practical method for grading the cognitive state of patients for the clinician. J. Psychiatr. Res..

[27] Yesavage J.A., Sheikh J.I. (1986). 9/Geriatric Depression Scale (GDS) recent evidence and development of a shorter violence. Clin. Gerontol..

[28] D’Ath P., Katona P., Mullan E., Evans S., Katona C. (1994). Screening, Detection and Management of Depression in Elderly Primary Care Attenders. I: The Acceptability and Performance of the 15 Item Geriatric Depression Scale (GDS15) and the Development of Short Versions. Fam. Pract..

[29] Almeida O.P., Almeida S.A. (1999). Short versions of the geriatric depression scale: A study of their validity for the diagnosis of a major depressive episode according to ICD-10 and DSM-IV. Int. J. Geriatr. Psychiatry.

[30] De Craen A.J.M., Heeren T.J., Gussekloo J. (2003). Accuracy of the 15-item geriatric depression scale (GDS-15) in a community sample of the oldest old. Int. J. Geriatr. Psychiatry.

[31] Underwood M., Lamb S., DiazOrdaz K., Ellard D.R., Potter R., Spanjers K., Taylor S.J.C., Eldridge S., Sheehan B., Slowther A. (2013). Exercise for depression in care home residents: A randomised controlled trial with cost-effectiveness analysis (OPERA). Health Technol. Assess..

[32] Underwood M., Eldridge S., Lamb S., Potter R., Sheehan B., Slowther A.-M., Taylor S., Thorogood M., Weich S. (2011). The OPERA trial: Protocol for a randomised trial of an exercise intervention for older people in residential and nursing accommodation. Trials.

[33] Jirak R., Kalvach Z., Zadak Z., Jirak R. (2004). Evaluation of psychological functioning in the elderly. Geriatrics and Gerontology.

[34] Vankova H. Current challenges in the field of geriatrics. Proceedings of the 25th Congress of Gerontology.

[35] Holmerova I., Vankova H., Hradcova D. The role of General Practitioner in Long Term Care of older adults. Proceedings of the 20th WONCA World Conference 2013: Family Medicine_Care for Generations.

[36] Chang C.-B., Chan D.-C. (2010). Comparison of Published Explicit Criteria for Potentially Inappropriate Medications in Older Adults. Drugs Aging.

[37] Brett J., Maust D.T., Bouck Z., Ignacio R.V., Mecredy G., Kerr E.A., Bhatia S., Elshaug A., Pearson S.A. (2018). Benzodiazepine Use in Older Adults in the United States, Ontario, and Australia from 2010 to 2016. J. Am. Geriatr. Soc..

[38] Lader M. (2012). Dependence and withdrawal: Comparison of the benzodiazepines and selective serotonin re-uptake inhibitors. Addiction.

[39] Beekman A.T., de Beurs E., van Balkom A.J., Deeg D.J., van Dyck R., van Tilburg W. (2000). Anxiety and depression in later life: Co-occurrence and communality of risk factors. Am. J. Psychiatry.

[40] Moscati A., Flint J., Kendler K.S. (2016). Classification of anxiety disorders comorbid with major depression: Common or distinct influences on risk?. Depress. Anxiety.

[41] Vink D., Aartsen M., Comijs H.C., Heymans M., Penninx B.W., Stek M.L., Deeg D.J., Beekman A.T. (2009). Onset of Anxiety and Depression in the Aging Population: Comparison of Risk Factors in a 9-Year Prospective Study. Am. J. Geriatr. Psychiatry.

[42] Kendall T., Cape J., Chan M., Taylor C. (2011). Guidelines: Management of generalised anxiety disorder in adults: Summary of NICE guidance. BMJ.

[43] Fick D.M., Cooper J.W., Wade W.E., Waller J.L., Maclean J.R., Beers M.H. (2003). Updating the Beers criteria for potentially inappropriate medication use in older adults: Results of a US consensus panel of experts. Arch. Intern. Med..

[44] Gallagher P., O’Mahony D. (2008). STOPP (Screening Tool of Older Persons’ potentially inappropriate Prescriptions): Application to acutely ill elderly patients and comparison with Beers’ criteria. Age Ageing.

[45] Cassel C.K., Guest J.A. (2012). Choosing wisely: Helping physicians and patients make smart decisions about their care. JAMA.

[46] Lankova J., Raboch J. (2013). Depression. Guidelines for General Practitioners. Deprese. Doporučený Postup pro Všeobecné Praktické Lékaře. Novelizace 2013.

[47] Cerveny R. (2014). Geriatrics. Guidelines for General Practitioners. Geriatrie. Doporučený Postup pro Všeobecné Praktické Lékaře. Novelizace 2014.

[48] Barreto P., Lapeyre-Mestre M., Mathieu C., Piau C., Bouget C., Cayla F., Vellas B., Rolland Y. (2013). Indicators of Benzodiazepine Use in Nursing Home Residents in France: A Cross-Sectional Study. J. Am. Med. Dir. Assoc..

[49] Vetrano D.L., Tosato M., Colloca G., Topinkova E., Fialova D., Gindin J., van der Roest H.G., Landi F., Liperoti R., Bernabei R. (2013). Polypharmacy in nursing home residents with severe cognitive impairment: Results from the SHELTER Study. Alzheimer’s Dement..

[50] Mhaoláin A.N., Gallagher D., O’Connell H., Chin A.-V., Bruce I., Hamilton F., Tehee E., Coen R.F., Coakley D., Walsh B. (2010). Benzodiazepine use amongst community dwelling elderly: 10 years on. Int. J. Geriatr. Psychiatry.

[51] Biering P. (2019). Helpful approaches to older people experiencing mental health problems: A critical review of models of mental health care. Eur. J. Ageing.

[52] Tannenbaum C., Martin P., Tamblyn R., Benedetti A., Ahmed S. (2014). Reduction of inappropriate benzodiazepine prescriptions among older adults through direct patient education: The EMPOWER cluster randomized trial. JAMA Intern. Med..

[53] Vicens C., Bejarano F., Sempere E., Mateu C., Fiol F., Socias I., Aragonès E., Palop V., Beltran J.L., Piñol J.L. (2014). Comparative efficacy of two interventions to discontinue long-term benzodiazepine use: Cluster randomised controlled trial in primary care. Br. J. Psychiatry.

[54] Paquin A.M., Zimmerman K., Rudolph J.L. (2014). Risk versus risk: A review of benzodiazepine reduction in older adults. Expert Opin. Drug Saf..

[55] Tannenbaum C. (2015). Inappropriate benzodiazepine use in elderly patients and its reduction. J. Psychiatry Neurosci..

[56] Martin P., Tamblyn R., Ahmed S., Tannenbaum C. (2013). A drug education tool developed for older adults changes knowledge, beliefs and risk perceptions about inappropriate benzodiazepine prescriptions in the elderly. Patient Educ. Couns..

[57] Bourgeois J., Elseviers M., Azermai M., Van Bortel L., Petrovic M., Stichele R.V. (2014). Barriers to discontinuation of chronic benzodiazepine use in nursing home residents: Perceptions of general practitioners and nurses. Eur. Geriatr. Med..

[58] Bourgeois J., Elseviers M.M., Van Bortel L., Petrovic M., Stichele R.H.V. (2014). Feasibility of discontinuing chronic benzodiazepine use in nursing home residents: A pilot study. Eur. J. Clin. Pharmacol..

[59] Souto Barreto P., Lapeyre-Mestre M., Cestac P., Vellas B., Rolland Y. (2016). Effects of a geriatric intervention aiming to improve quality care in nursing homes on benzodiazepine use and discontinuation. Br. J. Clin. Pharmacol..

[60] Leggett A., Kavanagh J., Zivin K., Chiang C., Kim H.M., Kales H.C. (2015). The Association between Benzodiazepine Use and Depression Outcomes in Older Veterans. J. Geriatr. Psychiatry Neurol..

[61] Morley J.E. (2014). Inappropriate Drug Prescribing and Polypharmacy Are Major Causes of Poor Outcomes in Long-Term Care. J. Am. Med. Dir. Assoc..

[62] Cestac P., Tavassoli N., Vellas B., Rolland Y. (2013). Improving Medication Use in the Nursing Homes: A European Perspective. J. Am. Med. Dir. Assoc..

